# Supplementary material for: Variational inference of single cell time series
Source: bioRxiv. 2024 Aug 30:2024.08.29.610389. Preprint. [Version 1] doi: 10.1101/2024.08.29.610389 (PMC11384007; doi:10.1101/2024.08.29.610389)
Supplement: Supplementary file 1 [file media-1.pdf]

8 Supplemental material

8.1 Deriving the variational lower bound

The count matrix $\mathbf{X}$ follows some complicated distribution $p(x)$, which is difficult to sample from. We aim to sample from a simpler distribution $p(z)$, found by maximizing $p(x)$ or the evidence lower bound (ELBO):

$$\begin{aligned}
\log p(x) &= \log \int_z p(x, z) dz \\
&= \log \int_z p(x, z) \frac{q(z|x)}{q(z|x)} dz \\
&= \log \mathbb{E}_{z \sim q(z|x)} \left[\frac{p(x, z)}{q(z|x)} \right] && \text{(A-1)} \\
&\geq \underbrace{\mathbb{E}_{z \sim q(z|x)} \left[\log \frac{p(x, z)}{q(z|x)} \right]}_{\text{evidence lower bound}}, && \text{(A-2)}
\end{aligned}$$

where the inequality is a consequence of Jensen's inequality. In practice, the variational posterior $q(z|x)$, is chosen such that it is easy to sample from. To quantify how it differs from the true posterior $p(z|x)$, one may compute the Kullback-Leibler divergence:

$$\begin{aligned}
D_{KL}\big(q(z|x)||p(z|x)\big) &= -\int_z q(z|x) \log \frac{p(z|x)}{q(z|x)} dz \\
&= -\int_z q(z|x) \log \frac{p(z, x)}{q(z|x)p(x)} dz \\
&= -\text{ELBO} + \log p(x). && \text{(A-3)}
\end{aligned}$$

Rearranging, we obtain:

$$\log p(x) = \text{ELBO} + D_{KL}\big(q(z|x)||p(z|x)\big). \tag{A-4}$$

Additionally, the ELBO can be further expanded:

$$\begin{aligned}
\text{ELBO} &= \int_z q(z|x) \log \frac{p(x,z)}{q(z|x)} dz \\
&= \int_z q(z|x) \log \frac{p(x|z)p(z)}{q(z|x)} dz \\
&= \int_z q(z|x) \log p(x|z) dz + \int_z q(z|x) \log \frac{p(z)}{q(z|x)} dz \\
&= \mathbb{E}_{z \sim q(z|x)} \big(\log p(x|z) \big) - D_{KL} \big(q(z|x) || p(z) \big). \qquad \text{(A-5)}
\end{aligned}$$

While KL divergence will be difficult to compute in most cases, closed form exists if $q(z|x) \sim \mathcal{N}(\mu_1, \Sigma_1)$ and $p(z) \sim \mathcal{N}(\mu_2, \Sigma_2)$:

$$D_{KL}\big(q(z|x)||p(z)\big) = \frac{1}{2} \left(\log \frac{|\Sigma_2|}{|\Sigma_1|} - n + \mathrm{Tr}(\Sigma_2^{-1}\Sigma_1) + (\mu_2 - \mu_1)^\top \Sigma_2^{-1} (\mu_2 - \mu_1) \right) \qquad \text{(A-6)}$$

By assuming $\mu_2 = \vec{0}$ and $\Sigma_2 = I$, the above expression can be further simplified to:

$$\begin{aligned}
D_{KL}\big(q(z|x)||p(z)\big) &= \frac{1}{2} \left(\log \frac{|I|}{|\Sigma_1|} - n + \mathrm{Tr}(I^{-1}\Sigma_1) + (\vec{0} - \mu_1)^\top I^{-1} (\vec{0} - \mu_1) \right) \\
&= \frac{1}{2} \Big(- \log |\Sigma_1| - n + \mathrm{Tr}(\Sigma_1) + \mu_1^\top \mu_1 \Big) \\
&= \frac{1}{2} \left(\log \prod_i \sigma_i^2 - n + \sum_i \sigma_i^2 + \sum_i \mu_i^2 \right) \\
&= \frac{1}{2} \left(\sum \log \sigma_i^2 - n + \sum_i \sigma_i^2 + \sum_i \mu_i^2 \right), \qquad \text{(A-7)}
\end{aligned}$$

where $|\cdot|$ indicates a determinant, and μ_i and σ_i are the ith elements of μ_1 and $\mathrm{diag}(\Sigma_1)$ respectively.

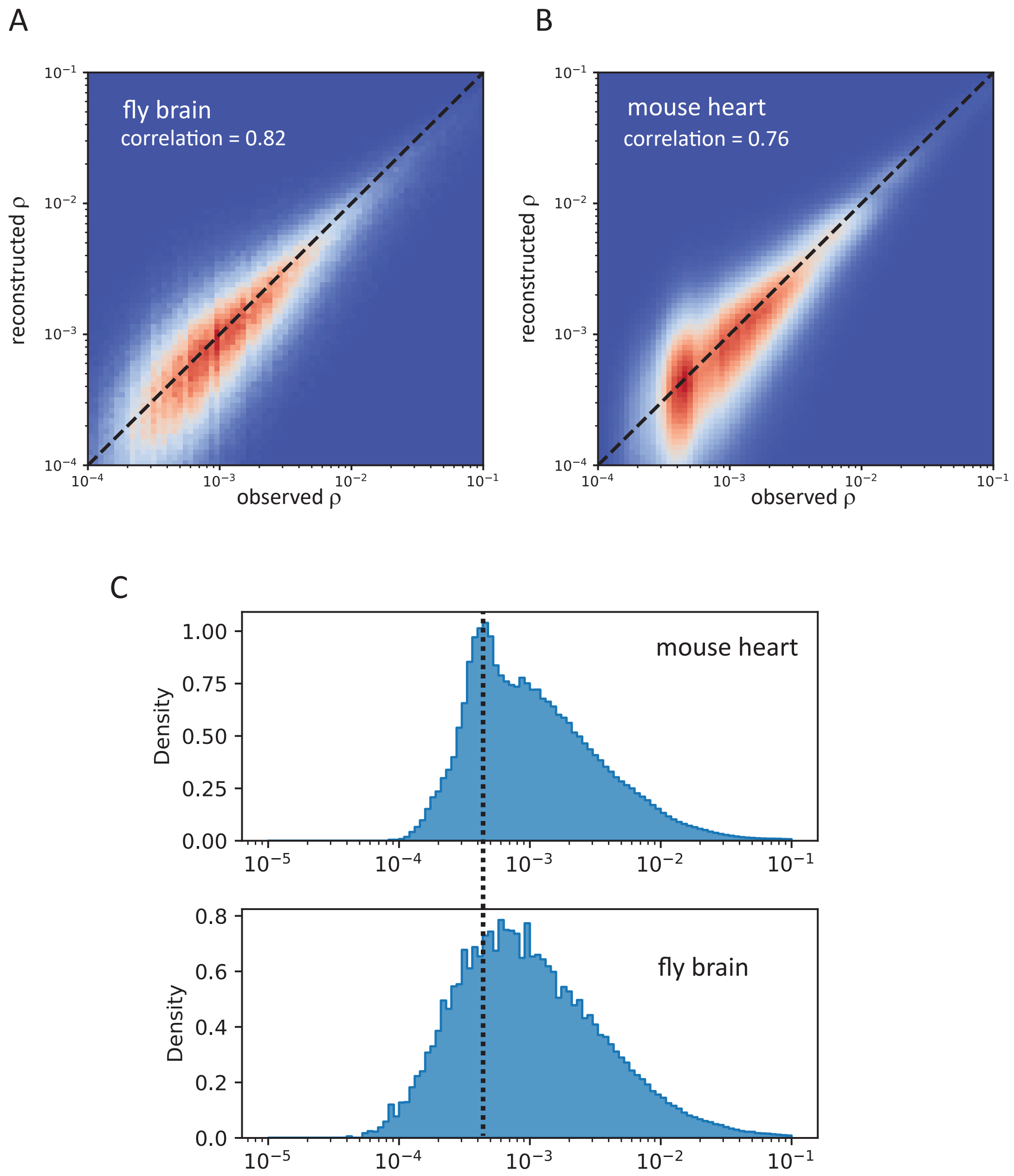

Figure S1: Relationship between the observed gene fraction and the reconstructed gene fraction for the clock neuron (A) and the mouse heart (B) dataset and their respective density (C).

A

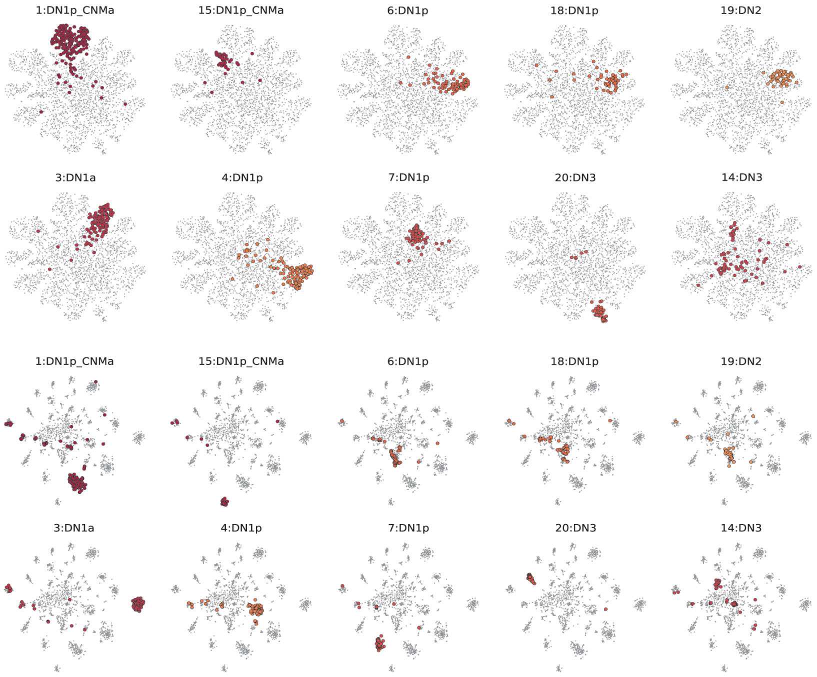

B

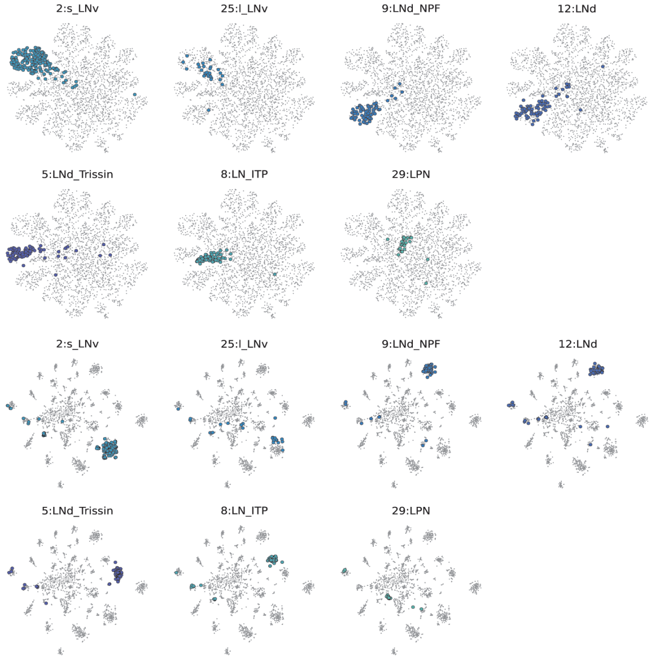

Figure S2: A: Comparison between SEURAT and SNOW embedding of the labeled dorsal neurons. B: Comparison between the SEURAT and SNOW embedding of the labeled lateral neurons.

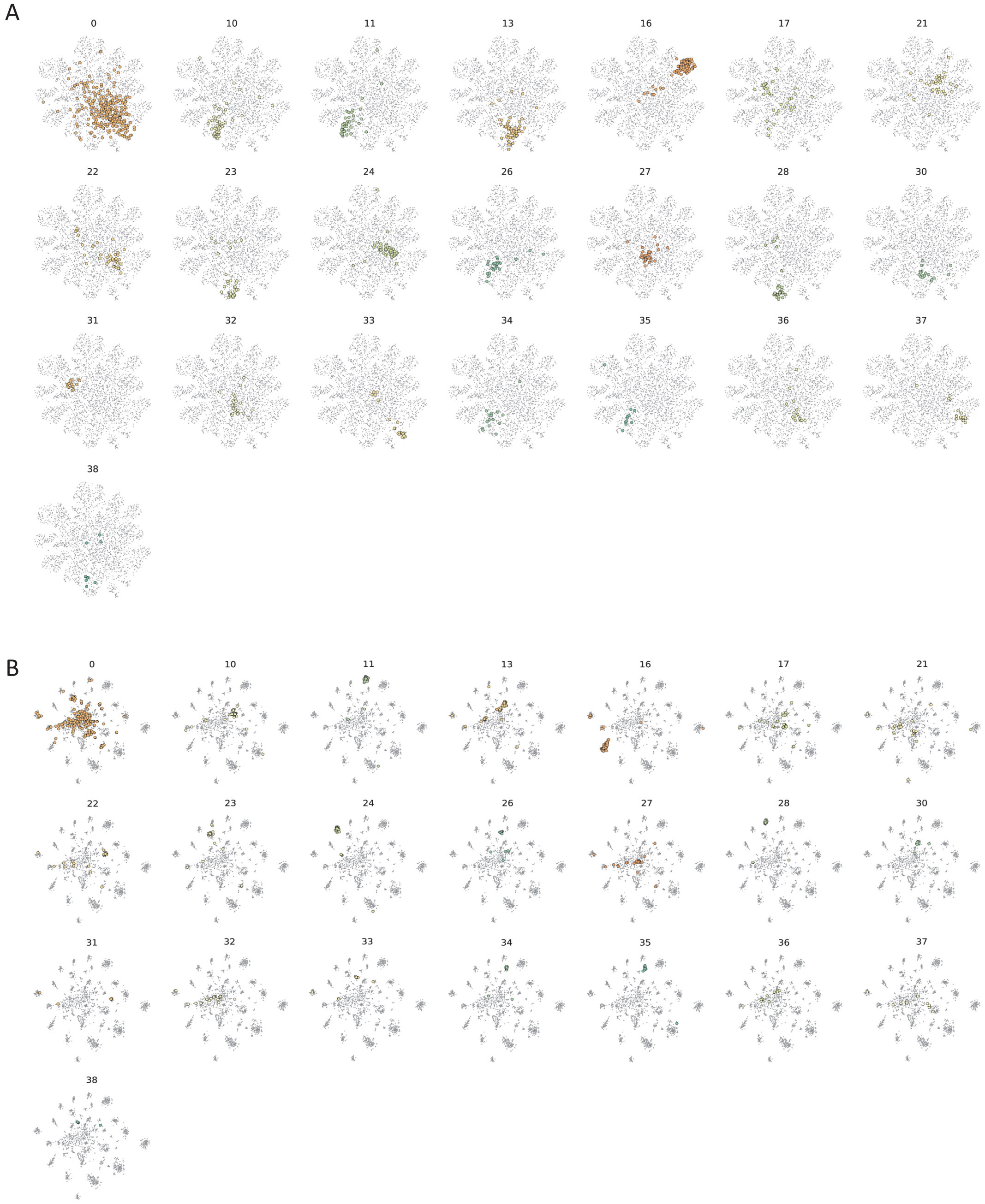

Figure S3: Comparison between the Seurat and snow embedding of unlabeled drosophila clock neurons.

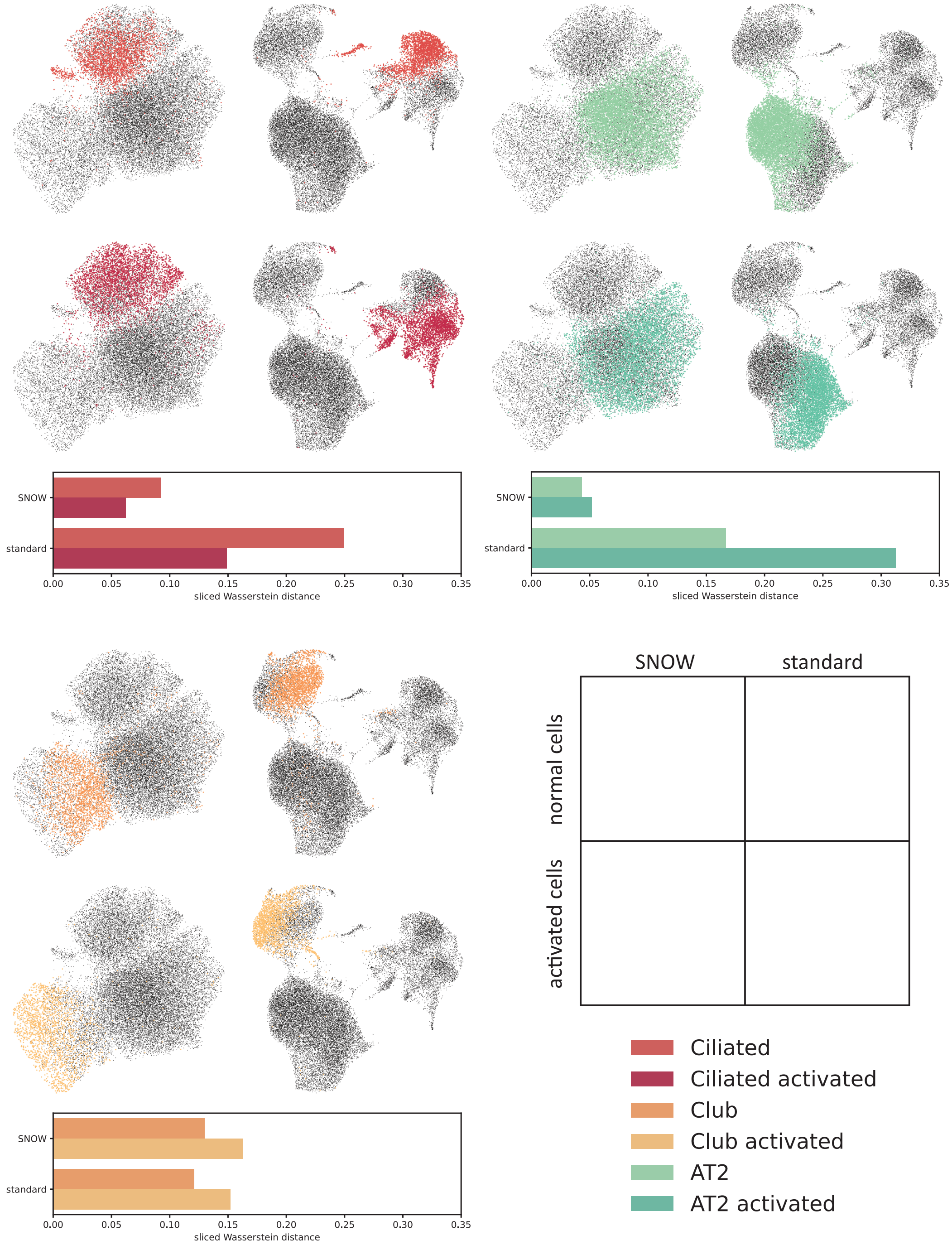

Figure S4: Comparison between the UAMP embedding produced with the raw data and with SNOW using the lung regeneration data. Bar chart below each panel shows the distance between each subtype and the main cell type it belongs to (for example, the distance between AT2 (subtype) and the combination of AT2 and AT2 activated). AT2: alveolar type 2 cells. The grid on the lower right illustrates the layout of each panel.

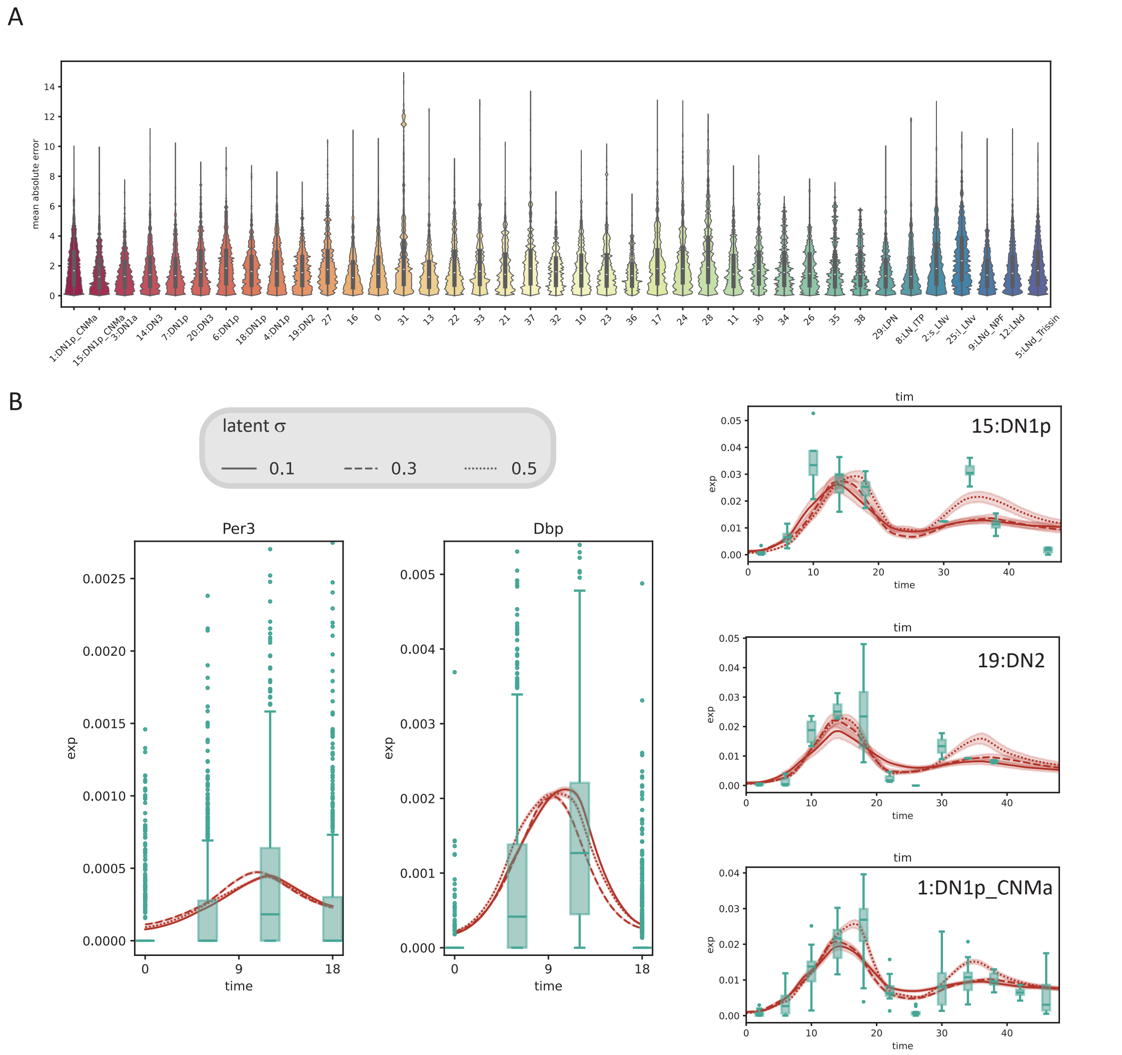

Figure S5: A: Mean absolute error of predicting time in the 38 neuron clusters from the clock neuron dataset. B: Impact of fixed latent space standard deviation on time series generation for the mouse heart (left) and the clock neuron dataset (right).

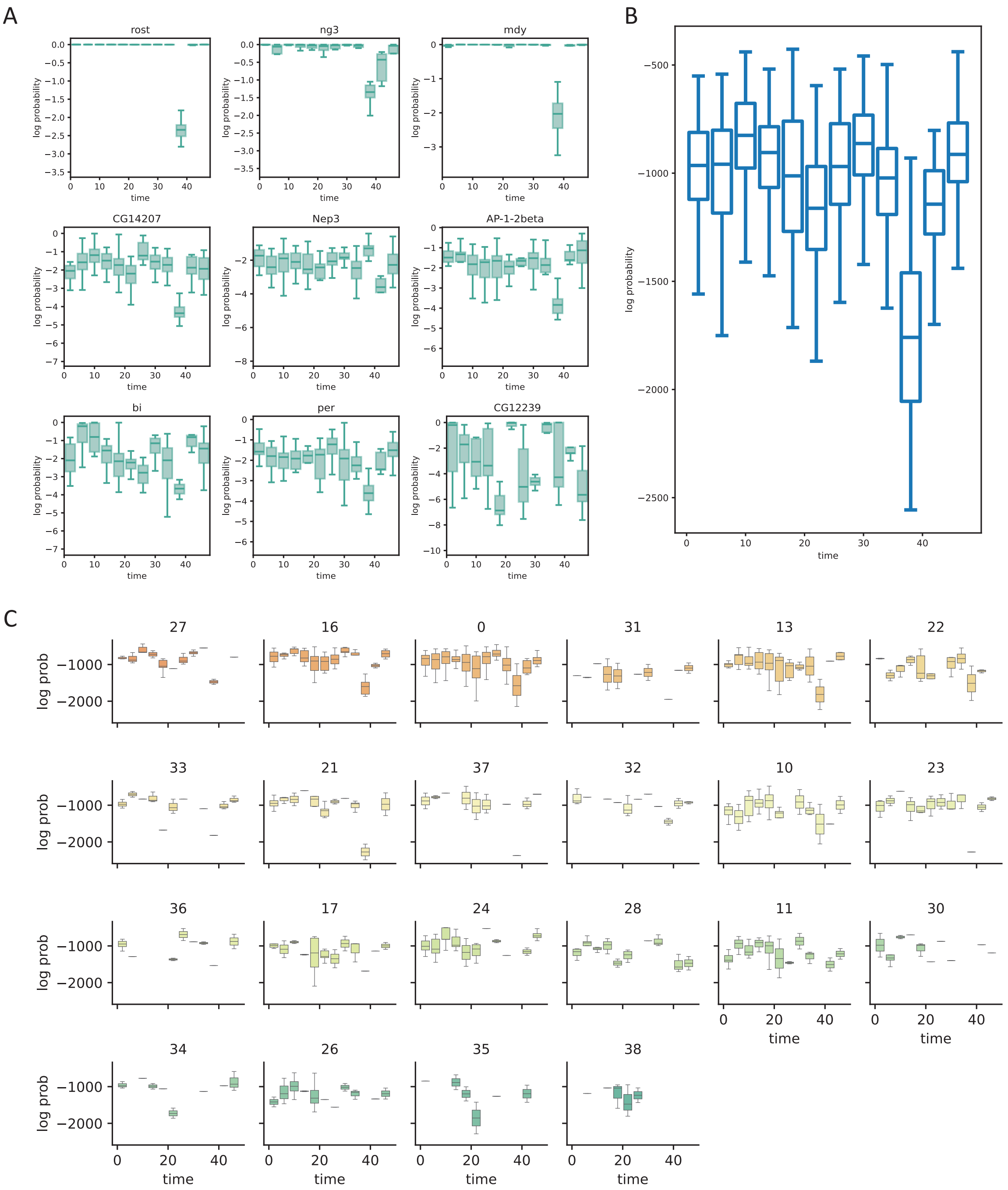

Figure S6: A: Box plot showing the log probability of observing each gene. B: Box plot showing the log probability of observing an entire cell. C: Box plot showing the log probability of observing each cell for the unnamed clusters.

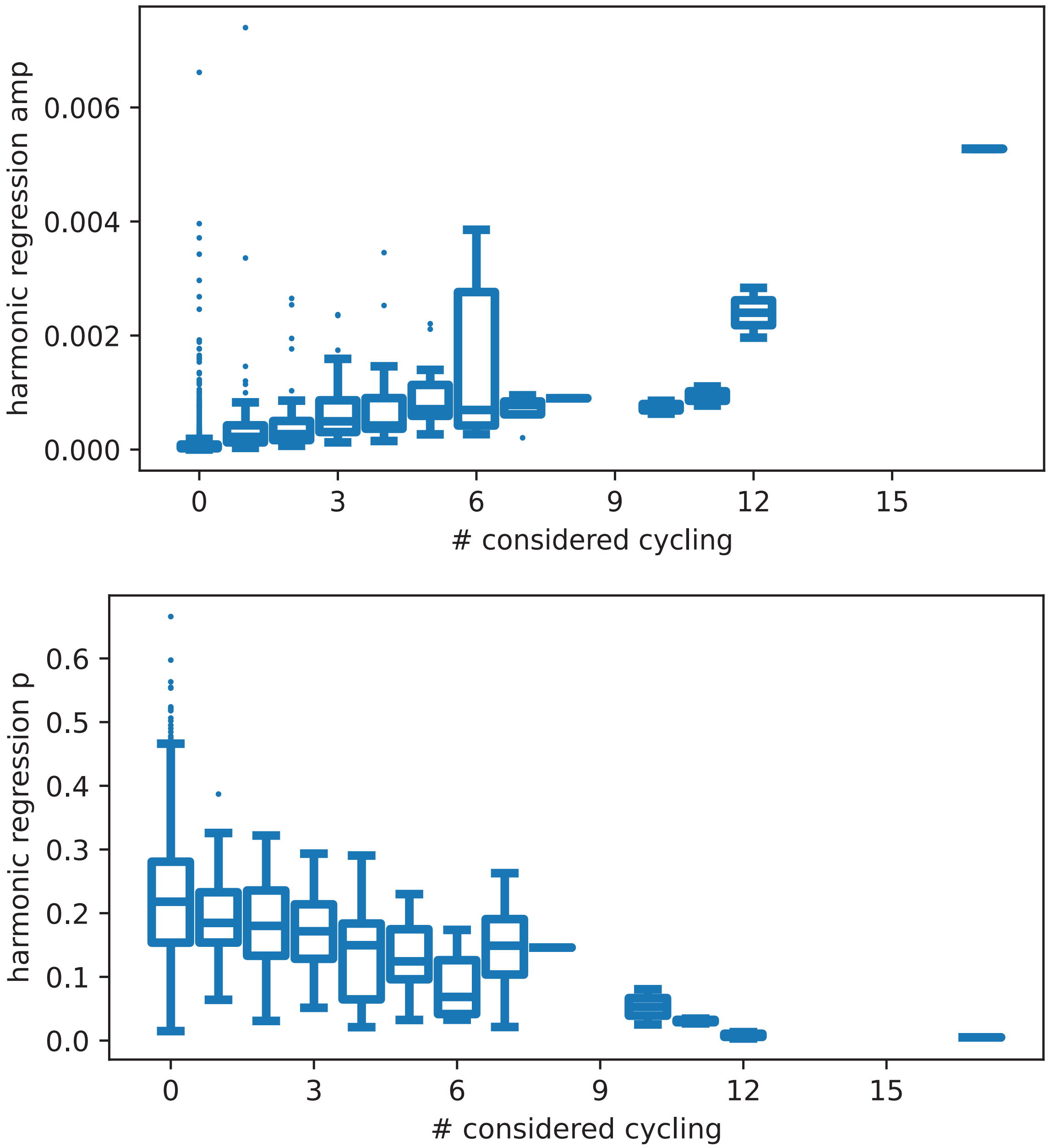

Figure S7: Top: Boxplot showing the relationship between the estimated amplitude and the number of time a gene is reported to be cycling by Ma et al. Bottom: Boxplot showing the relationship between the estimated p value and the number of time a gene is reported to be cycling by Ma et al.

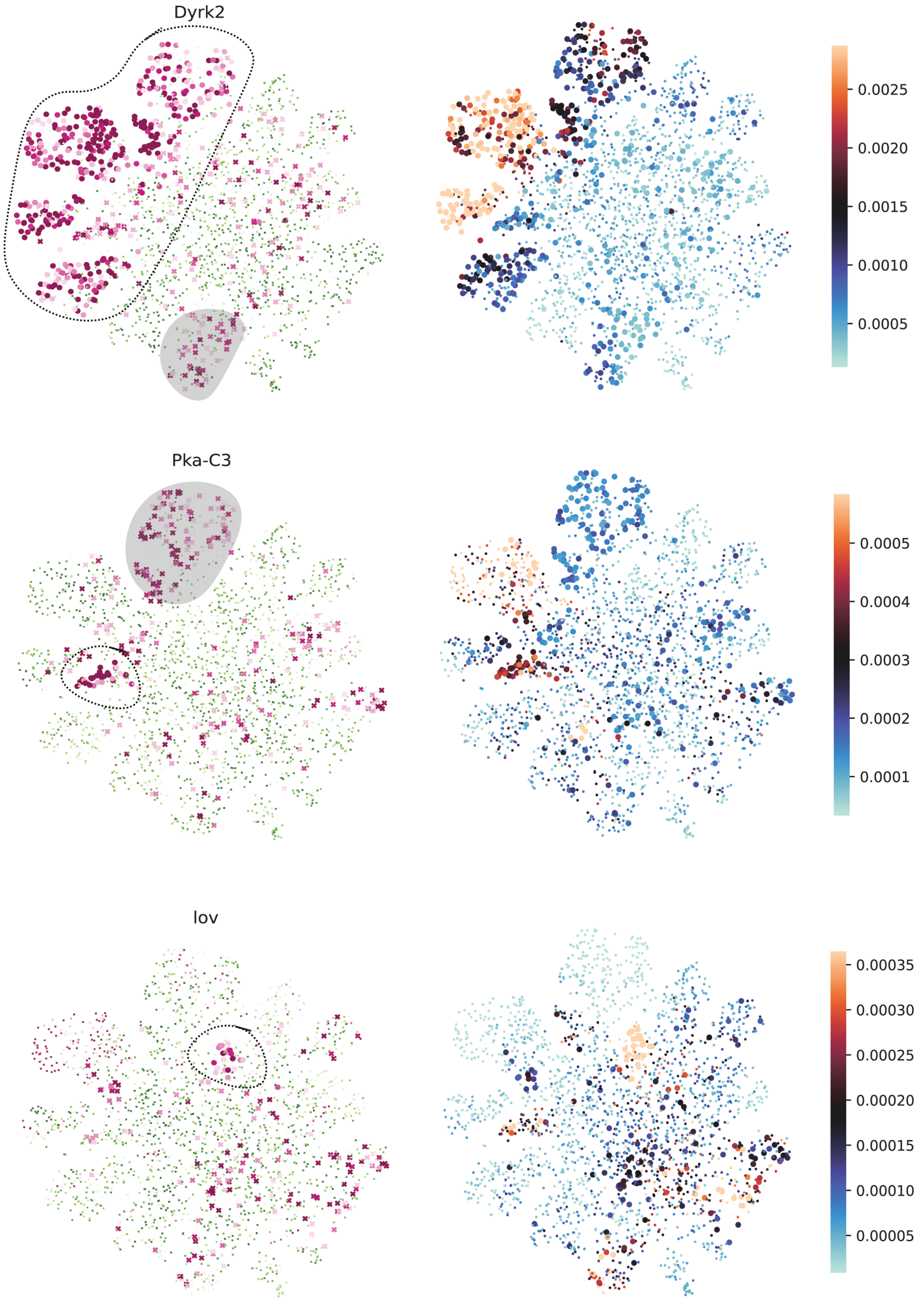

Figure S8: Example of genes that is reported to be cycling by Ma et al. Left column indicates estimated harmonic regression p value and right column the estimated amplitudes. Circled region indicates agreement between our analysis and that of Ma et al., and shaded region indcates disagreements. Cells with p-value greater than 0.001 or amplitude smaller than 0.0001 were made small for better visualization. Circular dots indicate that this gene is reported to be cycling in the cell type this cell belongs to, and vice versa for the crossed dots.

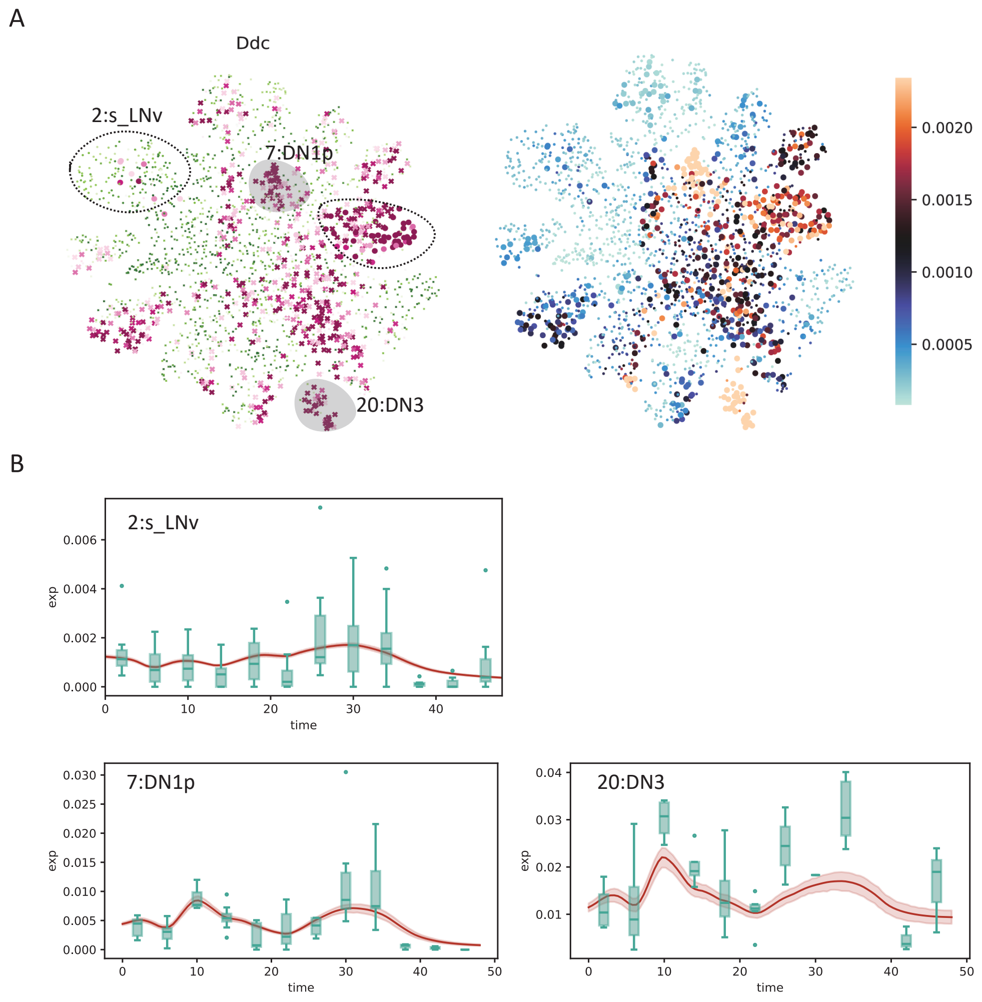

Figure S9: A: p value and amplitude of *Ddc* overlayed on top of the UMAP projection of the clock neuron data. B: SNOW generated time series (red lines) and experimental observation (green boxes) of cells within the circled and shaded region.
